# Chromatin Accessibility Reveals Adaptive Regulatory Variation in a Keystone Freshwater Fish

**DOI:** 10.1111/mec.70550

**Published:** 2026-09-21

**Authors:** Konrad Taube, Kristina Noreikiene, María‐Eugenia López, Riho Gross, Anti Vasemägi

**Affiliations:** ^1^ Estonian University of Life Sciences Tartu Estonia; ^2^ Vilnius University Vilnius Lithuania; ^3^ Swedish University of Agricultural Sciences Uppsala Sweden

## Abstract

Regulatory variation is increasingly recognized as a major driver of adaptive evolution, yet its role in natural populations remains largely unresolved. By integrating ATAC‐seq chromatin profiles with population genomic outlier scans and RNA‐seq gene expression data, we characterize the functional impact of regulatory elements and identify candidate loci where selection signatures coincide with tissue‐specific chromatin and transcriptional shifts. We mapped genome‐wide chromatin accessibility in three tissues (eye, liver and spleen) of wild European perch ( *Perca fluviatilis* ) inhabiting contrasting humic‐ (dark‐water) and clear‐water lakes. We identified over 76,000 accessible chromatin regions spanning 36.1 Mb of the genome, which were enriched for gene functions related to vision, metabolism and immune response. Chromatin landscapes were strongly tissue‐specific, while only a subset of regions (0.32%, *n* = 161) showed differential accessibility between environments, suggesting that the regulatory architecture is dominated by tissue identity rather than environmental context. Integrating population genomic outlier scans with chromatin profiles revealed significant enrichment of adaptive SNPs within common open chromatin regions (1.5‐fold) and transcription start sites (1.7‐fold), directly implicating cis‐regulatory elements as frequent targets of natural selection. This study demonstrates that humic adaptation in perch targets specific regulatory regions readily identified by the ATAC‐seq approach, highlighting the value of functional chromatin maps in evolutionary genomics. Together, our results provide one of the first genome‐wide regulatory maps for a wild vertebrate and establish a functional framework for connecting natural selection to regulatory mechanisms in the wild.

## 1 Introduction

Regulatory variation, which influences when, where and how genes are expressed, is widely recognized as a major driver of phenotypic evolution (King and Wilson 1975; Wray 2007; Carroll 2008; Siepel and Arbiza 2014; Sundaram and Wysocka 2020). In particular, *cis*‐regulatory elements such as promoters and enhancers play key roles in evolutionary divergence, especially in traits that vary across environments within species (Prud'homme et al. 2007; Villar et al. 2015; Zabidi and Stark 2016). Together, these elements form the regulatory landscape of the genome, shaping how organisms respond to internal and external cues (Ma and Zhang 2020; Grandi et al. 2022). While such non‐coding regions constitute a substantial portion of the genome, the functional consequences of sequence variation within specific regulatory elements remain to be fully characterized (Pavey et al. 2010; Wellenreuther and Hansson 2016; Dhamija and Menon 2018). Thus, identifying regulatory regions under selection and linking them to specific functional mechanisms represents a central challenge in understanding the genetic basis of adaptive evolution.

The regulatory landscape exerts its influence on gene expression in part through the chromatin environment. When chromatin is in an open state, transcription factors and other regulatory proteins can access DNA and initiate transcription (Klemm et al. 2019). A powerful method for genome‐wide profiling of open chromatin is ATAC‐seq (assay for transposase‐accessible chromatin using sequencing), which uses a hyperactive transposase to insert sequencing adapters into nucleosome‐free regions, thereby identifying potential regulatory elements such as promoters or enhancers (Buenrostro et al. 2013, 2015; Yan et al. 2020). ATAC‐seq has been increasingly applied in model organisms to study chromatin state and transcriptional regulation (Davie et al. 2015; Grbesa et al. 2017; Cusanovich et al. 2018; Liu et al. 2019; Jiang et al. 2022; Pettie et al. 2024; Yang et al. 2025), but its use in wild populations remains limited. Recent studies have started to bridge this gap by applying ATAC‐seq to natural populations to map chromatin variation associated with ecological or environmental divergence. These studies show, for instance, that accessibility patterns can differ between populations inhabiting distinct habitats, potentially reflecting environmentally induced plasticity or regulatory divergence (Weizman and Levy 2019; Villarroel et al. 2021; Weizman et al. 2021; Husby 2022; Shen et al. 2023; Luo et al. 2025). However, few studies have directly linked chromatin accessibility to population genomic signals of selection, leaving unresolved questions about how regulatory variation contributes to adaptation in the wild (Krishnan et al. 2022; Tian et al. 2024).

Populations exposed to ecologically challenging or even extreme conditions are highly suitable for elucidating the importance of regulatory variation in adaptation (Xin et al. 2020; Weizman et al. 2021; Hu et al. 2022; Yan et al. 2024). The Eurasian perch ( *Perca fluviatilis* ) in humic (dark‐water) lakes serves as a particularly relevant teleost model for studying the significance of the regulatory landscape in local adaptation. Perch in humic lakes are exposed to rather extreme conditions, characterized by low pH, reduced light penetration and hypoxic conditions (Jones 1992; McKnight and Aiken 1998). Despite these harsh conditions, the perch is one of the few temperate fish species that thrives in humic water bodies, making it a valuable model for studying the contribution of regulatory variation to population‐level divergence (Ozerov et al. 2022, 2025; Noreikiene et al. 2024; López et al. 2025). Phylogeographic studies have indicated that European perch lineages underwent relatively recent demographic changes during the Pleistocene, including contractions associated with glacial cycles and subsequent postglacial expansion from multiple refugia (Nesbø et al. 1999; Costedoat and Gilles 2009). Following the retreat of the last continental ice sheets, northern European freshwater systems were recolonized approximately during the early Holocene (~10,000–12,000 years ago), with subsequent geographic structuring among drainage systems and freshwater populations (Nesbø et al. 1999). Importantly, northern European perch populations, particularly within the Baltic Sea region, represent zones where divergent postglacial lineages have persisted and admixed, contributing to complex population structure and elevated genetic diversity (Lichman et al. 2024, 2026). These patterns indicate that present‐day perch have been shaped by both historical colonization processes and ongoing interactions among populations, in addition to habitat‐specific selection pressures. Although the relatively recent colonization history suggests that contemporary responses may involve both plasticity and evolutionary adaptation, restricted connectivity and contrasting selection pressures among habitats have enabled measurable population differentiation and local adaptation (Ozerov et al. 2022; López et al. 2025). Previous whole‐genome analyses of perch populations inhabiting these contrasting habitats have identified hundreds of candidate loci under selection, particularly in genes related to immune function, ion transport and neuronal development, highlighting that adaptation to humic environments influences multiple physiological processes (Ozerov et al. 2022, 2025; López et al. 2025). Importantly, selection signals are enriched in regulatory regions, such as 5′ and 3′ untranslated regions (UTRs), underscoring the contribution of regulatory evolution to adaptive divergence (Wray 2007; Naidoo et al. 2018). However, the contribution of distal non‐coding regulatory elements, such as open chromatin regions, to these adaptive patterns remains largely unexplored.

Here, we use ATAC‐seq to map genome‐wide chromatin accessibility in eye, liver and spleen tissues from perch inhabiting humic‐ and clear‐water lakes. Our first objective was to characterize chromatin accessibility variation across tissues and environments, focusing on functional processes hypothesized to be involved in visual function, metabolism and immune response (Ozerov et al. 2022). Chromatin accessibility is known to vary at multiple biological scales, including among individuals and due to genetic and regulatory divergence (Degner et al. 2012; Moreno and Acar 2022). However, based on fundamental biological differences between tissues, we predicted that accessibility patterns would primarily reflect tissue‐specific functions rather than population or environmental differences, consistent with findings in other species (Xin et al. 2020; Hu et al. 2022). This hierarchical expectation provides a framework to first account for tissue‐specific regulatory programs and then test for finer‐scale signals of adaptive divergence. Secondly, we assessed the genomic distributions of accessible chromatin regions across annotation categories, testing for enrichment in transcription start sites (TSS) and promoter regions as analytical starting points to both validate our data and categorize peak regions for downstream testing of environment‐specific regulatory differences. We also characterized the types of transcription factor binding motifs—short DNA sequences that serve as binding sites for regulatory proteins and play a crucial role in modulating gene expression in response to environmental and physiological demands (Latchman 1997; Spitz and Furlong 2012)—present in accessible chromatin regions. Rather than testing whether motifs occur, this analysis provides an analytical starting point to further describe the regulatory landscape. Third, we assessed whether highly divergent outlier SNPs associated with humic adaptation (López et al. 2025) were enriched in open chromatin regions, which would suggest a regulatory basis for adaptive divergence. Finally, we evaluated whether integrating chromatin accessibility and outlier regions with other genomic datasets, such as eQTLs and differentially expressed genes (DEGs), can reveal putative regulatory mechanisms contributing to adaptation. By combining ATAC‐seq with recently published population genomic (López et al. 2025) and transcriptomic (Ozerov et al. 2025) datasets, our study provides an important step towards connecting non‐coding regulatory elements with adaptive divergence. More broadly, this work illustrates the value of multi‐layered genomic approaches for uncovering molecular targets of evolutionary change and advancing our understanding of the regulatory basis of local adaptation.

## 2 Materials and Methods

### 2.1 Sample Collection and Preparation

A total of six female *P. fluviatilis* individuals were collected from three clear‐water lakes (Äntu Valgejärv, Viitna Pikkjärv, and Piigandi) and three humic‐water lakes (Kuulma, Heisri Mustjärv, and Matsimäe Pühajärv) in Estonia in August 2021 using gillnets and rods (Figure S1, Table S1). We sampled across multiple lakes to minimize population‐specific effects and detect patterns associated with tissue identity and water type. Fish were euthanized with an overdose of benzocaine and measured for wet weight and total length. Multiple tissues (left whole eye, liver, spleen and left gill arch) were dissected from each fish, and immediately snap‐frozen on dry ice and stored at −80°C. Tissue selection focused on key functional processes hypothesized to be central to local adaptation: vision (eye), metabolism (liver), osmoregulation (gill) and immune response (spleen). Samples were then shipped on dry ice to Active Motif (California, USA) for library construction and sequencing using an Illumina Nextseq 500 with paired‐end 42 bp reads (PE42), targeting a minimum sequencing yield of 30 million reads per sample. Pilot sequencing indicated high‐quality libraries for the eye, liver and spleen, whereas gill libraries failed to meet prespecified quality‐control criteria established by Active Motif (data not shown). Consequently, only the three tissues that passed quality control were included for full‐scale library preparation and sequencing.

### 2.2 Read Processing and Alignment

Read quality was evaluated using FastQC v0.11.8 (Andrews 2010). Adapters, base calling errors and low‐quality sequences (Phred score < 30) were removed using Cutadapt (Martin 2011). Cleaned reads were aligned to the *P. fluviatilis* reference genome (GCF_010015445.1) (Roques et al. 2020) using Bowtie2 v2.4.4 with the following parameters ‐p 8 ‐D 20 ‐R 3 ‐N 0 ‐L 20 ‐i S,1,0.50 (Langmead and Salzberg 2012). PCR duplicates were removed using Picard v3.1.0 MarkDuplicates (https://broadinstitute.github.io/picard/), unpaired reads were filtered out using BamTools (Barnett et al. 2011) and multimapped reads were filtered using samtools v1.10 (Li et al. 2009). The resulting alignments were subsequently analysed with the multiBamSummary tool from the deepTools suite (Ramírez et al. 2016).

### 2.3 ATAC‐Seq Peak Calling Analysis

ATAC‐seq peaks were called using MACS2 v.2.1.0 (Zhang et al. 2008) with the ‐nomodel option and a *p*‐value cutoff of 1e^−7^. A consensus peak set was generated from individual peak files using the *mergePeaks* function in HOMER (v5.1; Heinz et al. 2010). For downstream analyses, RPKM‐normalized bigWig files were created using the bamCoverage tool within the deepTools suite (v3.5.6) and used to assess sample similarity and chromatin accessibility profiles. Finally, fragment size distributions were evaluated using the R package ATACseqQC (v1.15.8) (Ou et al. 2018) to ensure high‐quality transposition.

### 2.4 Peak Annotation and Gene Ontology Analysis

Consensus peaks were annotated with the HOMER tool *annotatePeaks.pl* using default parameters. Peak annotations were assigned based on genomic coordinates relative to the *P. fluviatilis* reference genome annotation, with HOMER assigning each peak to the nearest annotated genomic feature. Therefore, annotation categories represent standardized genomic classifications rather than experimentally defined regulatory boundaries. Regions were classified as intergenic, intronic, exonic, promoter‐TSS or genomic regions. Additionally, we annotated genomic regions predicted to bind non‐canonical, three‐stranded nucleic acid structures involved in gene regulation (i.e., triplex target site; TTS). To test whether accessibility was preferentially located in first exons or introns, we fitted a generalized linear mixed‐effects model (GLME) with a binomial error distribution, a logit link function and Laplace approximation to examine the probability of peaks occurring in the first exon or intron relative to the total number of exons or introns within a gene. Gene ID was included as a random effect to account for multiple peaks per gene. Models were run in RStudio (R version 4.4.0) using the lme4 package (Bates et al. 2015); predictions and visualization were carried out with ggpredict (Lüdecke 2018) and ggplot2 (Wickham 2016).

Gene ontology (GO) analysis was performed utilizing human orthologs of *P. fluviatilis* genes in ShinyGO 0.81 (Ge et al. 2019). We chose to use human orthologs because human genes have the most comprehensive and deeply curated annotations, allowing higher functional resolution. Pathways were determined to be enriched if the false discovery rate (FDR, the expected proportion of false positives among significant results) was < 0.01.

### 2.5 Association Between Chromatin Accessibility and Gene Expression

To analyse the relationship between chromatin accessibility and gene expression, we performed a gene‐level overlap analysis using tissue‐specific ATAC‐seq peak files. Although peaks were previously annotated with HOMER, which assigns peaks to the nearest gene, we generated a separate dataset based on direct genomic overlap. This approach avoids nearest‐gene assignment and ensures that only genes with direct chromatin accessibility signals were included. Genes overlapping at least one peak were recorded for each tissue.

For this analysis, promoter regions were operationally defined as a ±1 kb window surrounding annotated transcription start sites (TSSs) to capture core promoter sequences together with immediately adjacent proximal regulatory regions. Gene expression data for the eye, liver and spleen were obtained from Ozerov et al. (2025), including both DEGs (adjusted *p*‐value ≤ 0.05) and expressed genes that were not differentially expressed between humic‐ and clear‐water populations. In that study, 29 and 40 perch were sampled from eight humic‐ and eight clear‐water lakes in Estonia, respectively, representing 16 wild Eurasian perch populations. Genes were classified as expressed or non‐expressed based on a threshold of 10 reads per gene, with genes having < 10 reads considered non‐expressed.

These data were used in two complementary analyses. First, the association between chromatin accessibility and gene expression status (i.e., expressed versus non‐expressed) was evaluated using 2 × 2 contingency tables comparing gene expression category and ATAC‐seq peak presence or absence, with significance assessed using Fisher's exact test. Second, to evaluate whether differential gene expression between humic‐ and clear‐water environments is associated with chromatin accessibility, DEGs were compared to non‐DEGs, and ATAC‐seq peaks were further classified as variable (present in 1–5 of six individuals) or non‐variable (present in all six individuals), with significance assessed using Fisher's exact test.

### 2.6 Motif Analysis

To identify transcription factor (TF) binding motifs, we performed de novo motif discovery using HOMER's *findMotifsGenome.pl* (Heinz et al. 2010). Enriched motifs (using a *p*‐value threshold of < 1e^−50^, as suggested by HOMER) were subsequently annotated with *annotatepeaks.pl*. In addition to motif enrichment, we report mean motif scores, which reflect the average motif match strength; higher values indicate stronger similarity and binding affinity to the canonical TF binding motif (Grant et al. 2011).

### 2.7 Differential Peak Accessibility Analysis

To identify differentially accessible regions between humic‐ and clear‐water environments and among tissues (eye, liver and spleen), we performed differential analysis using edgeR v3.21 (Robinson et al. 2010). Read counts per peak were obtained by converting peak coordinates into .saf format and quantifying overlapping reads with featureCounts v2.1.1 (Liao et al. 2014). This generated a count matrix of accessibility per peak region, which served as input for differential analysis in edgeR. Separately, we used binomial generalized linear mixed‐effects models fitted with *glmer* to test whether gene architecture influenced peak localization. First‐exon or first‐intron overlap was modelled as a function of the total number of exons or introns in the corresponding gene, with gene identity included as a random intercept to account for multiple peaks associated with the same gene. No interaction terms were included because this analysis focused on the main effect of gene architecture on peak localization. Peaks with an FDR < 0.05 and an absolute |log_2_FC| > 1 were classified as differentially accessible.

### 2.8 Enrichment of Selection Signals in Open Chromatin Regions

To test whether signatures of natural selection are enriched in regions of open chromatin, we examined the overlap between ATAC‐seq peaks and SNPs previously identified as candidates under selection. Outlier SNPs were obtained from López et al. (2025), who analysed genome‐wide variation using a Pool‐seq whole‐genome sequencing (WGS) approach across 42 perch populations (22 humic‐ and 20 clear‐water lakes). In that study, signatures of selection were identified using a composite selection signal (CSS) approach that integrates allele frequency differentiation and genotype–environment associations. SNPs with Benjamini–Hochberg‐adjusted *q*‐values < 0.05 were considered candidates under selection, resulting in a total of 3556 outlier SNPs from approximately 900,000 filtered variants. To assess enrichment, outlier SNPs were intersected with ATAC‐seq peak regions and categorized based on peak annotations (including tissue‐specific peak sets and functional categories such as promoter‐TSS and exonic regions). Enrichment of outlier SNPs within these categories was then evaluated relative to their genomic distribution. The frequency of outlier SNPs within each peak category was compared with the frequency expected from the full set of approximately 900,000 filtered SNPs included in the original selection scan, thereby accounting for the underlying genomic distribution of tested variants.

Pearson's Chi‐squared tests with Yates' continuity and multiple testing (Bonferroni) correction were applied to test for significant enrichment. To further investigate how the frequency of open chromatin regions across individuals relates to potential signals of selection, we classified peaks based on their prevalence among samples, rather than by tissue identity alone. Specifically, across all samples, we grouped peaks into three categories: those observed in only 1–2 samples (rare peaks), 3–9 samples (non‐rare peaks), and 10–18 samples (common peaks). The cutoffs were chosen to distinguish between sample‐specific variation emerging from a lack of technical replications and broadly conserved regulatory regions.

### 2.9 Integrative Omics and Candidate Region Analysis

To assess the potential of integrating multiple omics datasets for identifying targets of natural selection, we combined our ATAC‐seq data with published Pool‐seq; RNA‐seq and eQTL datasets for a subset of candidate regions influenced by humic adaptation in perch (López et al. 2025; Ozerov et al. 2025). Specifically, Ozerov et al. identified over 5000 differentially expressed genes (DEGs) between humic‐ and clear‐water populations, highlighting the broad physiological impact of the humic environment on perch physiology and allowing for the prioritization of candidate genes and putative SNP‐gene expression associations. In this integrative framework, outlier SNPs were used to identify genomic regions with elevated population differentiation, consistent with selection, whereas ATAC‐seq peaks were used to assess whether these candidate regions overlapped accessible chromatin with potential regulatory activity. Differentially expressed genes provided evidence for environment‐associated transcriptional responses, while *cis*‐eQTLs allowed us to evaluate whether genetic variants within these regions were associated with variation in gene expression, providing a potential mechanistic link between sequence variation and regulatory effects.

*Cis*‐eQTLs were defined by Ozerov et al. (2025) as significant associations between SNP genotype and the expression level of nearby genes (FDR ≤ 0.05), identified using linear models within a *cis*‐regulatory window extending ±50 kb from gene boundaries. For integrative analyses, we examined genomic regions previously identified as under selection by Ozerov et al. (2025) (based on *q* < 0.05 and the presence of ≥ 3 outlier SNPs within 50 kb) and evaluated whether these regions overlapped ATAC‐seq peaks, differentially expressed genes (FDR ≤ 0.05) and/or significant *cis*‐eQTLs. Representative regions were selected based on prior identification of candidate genes under selection and the presence of at least one additional regulatory signal (e.g., eQTL, DEG and/or clustered outlier SNPs within an ATAC‐seq peak, providing biologically meaningful support for shared regulatory mechanisms underlying environmental adaptation). Manhattan plots were generated in Graphpad Prism 10.4.0 and genome track visualizations of these regions were produced with JBrowse 2 (The JBrowse Consortium 2023).

## 3 Results

**FIGURE 1:**
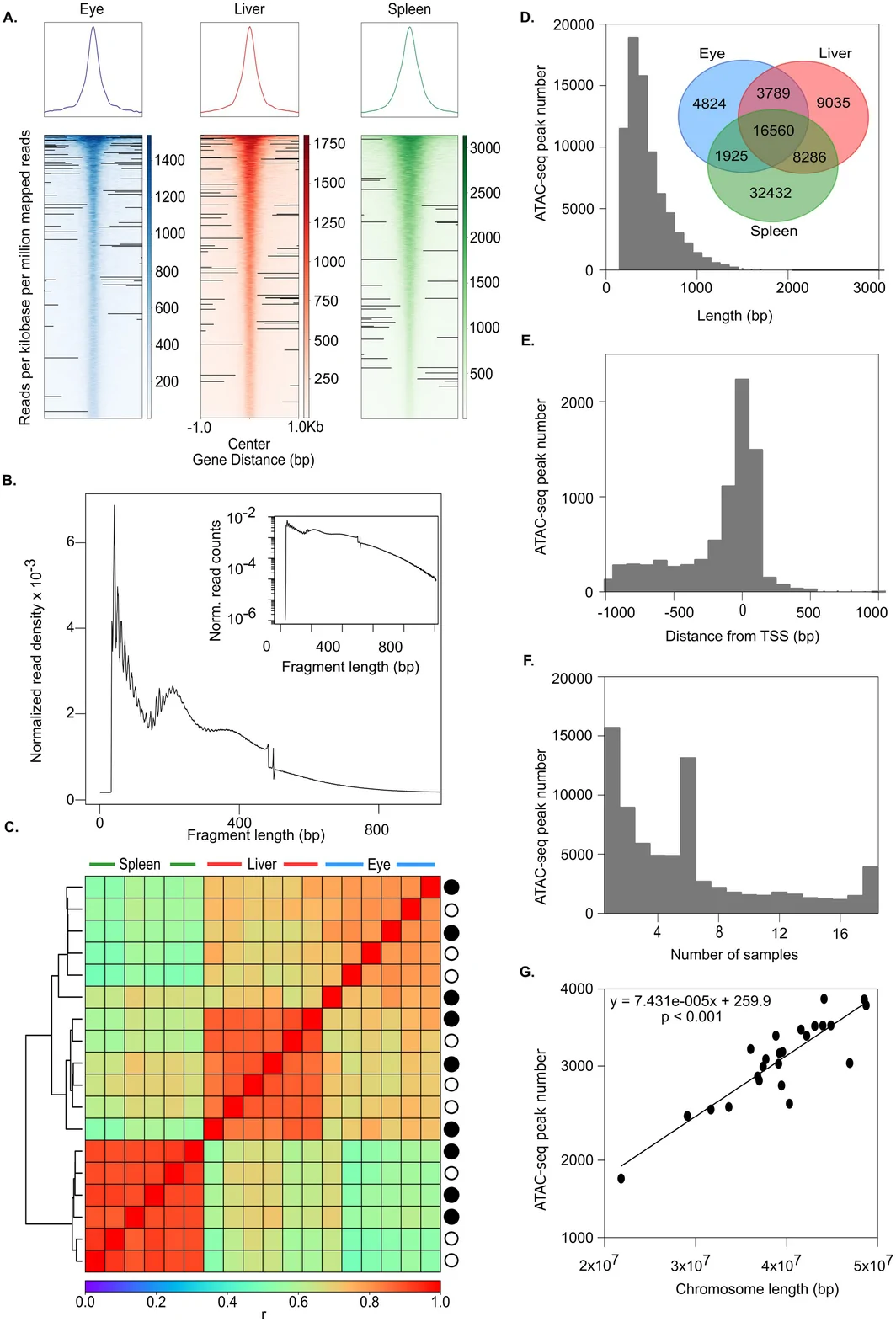
Characteristics of ATAC‐seq peaks across eye, liver and spleen tissues in *Perca fluviatilis* . (A) Patterns of chromatin accessibility (*n* = 76,850) across eye (blue), liver (red) and spleen (green) samples, showing tissue‐specific differences. Gene distance (bp) is measured from the TSS. Sparse horizontal black bars signify genomic intervals that are inaccessible (lacking ATAC‐seq signal). (B) Distribution of DNA fragment lengths in a representative spleen sample, reflecting nucleosome position (normalized counts shown in inset). (C) Sample clustering of ATAC‐seq peaks by tissue type and habitat; unfilled and filled circles represent samples from clear‐ and humic‐water environments, respectively. (D) Distribution of ATAC‐seq peak lengths, including a Venn diagram of shared and tissue‐specific peaks. (E) Distribution of peak distances relative to promoter–TSS sites. (F) Number of ATAC‐seq peaks detected as a function of the number of studied contributing. (G) Relationship between chromosome size (Mb) and the number of identified ATAC‐seq peaks.

Analysis of 18 samples from three humic‐ and three clear‐water lakes generated between 40.3–55.1 M raw reads and 34.0–52.1 M filtered reads per sample (Table S1). After quality control, between 9411 and 47,678 ATAC‐seq peaks were detected per sample. The resulting consensus peak set comprised 76,850 chromatin accessibility peaks, ranging from 199 to 3036 base pairs (bp) in length (Table S2). These peaks collectively covered 3.80% of the 951.3 Mb Eurasian perch genome. Within a 5 kb upstream or downstream of genes, a total of 11,627 (eye), 14,206 (liver) and 20,049 (spleen) genic regions (i.e., exon, intron and promoter TSSs) overlapped with ATAC‐seq peaks in eye, liver and spleen tissue, respectively. The spleen showed consistently higher levels of chromatin accessibility compared to the other tissues, with averages of 13,768 (eye), 24,600 (liver) and 46,489 (spleen) ATAC‐seq peaks (Table 1; Figure 1A,D).

**TABLE 1:** Summary of ATAC‐seq dataset comprising eighteen *P. fluviatilis* samples from three tissues, collected from humic‐ and clear‐water lakes.

Environment	Lake	Coordinates	No. of ATAC‐seq peaks		
			Whole eye	Liver	Spleen
Clear	Äntu Valgejärv	59.060, 26.241	10,597	22,590	35,534
Clear	Viitna Pikkjärv	59.444, 26.011	9411	28,629	39,920
Clear	Piigandi	58.013, 26.784	16,141	26,854	45,172
Humic	Matsimäe Pühajärv	59.061, 25.514	11,963	27,773	40,549
Humic	Kuulma	57.956, 27.163	11,615	20,806	47,678
Humic	Heisri Mustjärv	58.027, 26.828	22,886	20,953	40,084
*Merged consensus averages*			13,768	24,600	41,489

Initial heatmaps for each tissue revealed clear tissue‐specific differences in read density (Figure 1A). The ATAC‐seq fragment length distribution followed a characteristic pattern with a predominance of short fragments (< 200 bp), representing nucleosome‐free regions, and progressively fewer longer fragments (≥ 200 bp) corresponding to mono‐, di‐ and tri‐nucleosomes, confirming expected chromatin digestion patterns (Figure 1B). The majority of ATAC‐seq peaks were short (< 500 bp) and rarely exceeded 1000 bp. A global heatmap of all samples showed distinct clustering by tissue type (Figure 1C). While 21.5% of peaks were shared across all three tissues, the spleen contained a proportionally higher number of unique peaks (70.1%) compared to eye and liver (Figure 1D, Venn diagram). As expected, the majority of TSS‐associated peaks occurred at the start of the transcription starting site or slightly upstream (5.59%, Figure 1E). Peaks were often detected in groups of six, twelve or eighteen samples, reflecting consistent tissue‐dependent presence or absence (Figure 1F). Finally, the number of ATAC‐seq peaks per chromosome showed a strong positive correlation with chromosome size (Figure 1G, *r* ^2^ = 0.47).

### 3.1 Annotation and Gene Ontology of ATAC‐Seq Data

**FIGURE 2:**
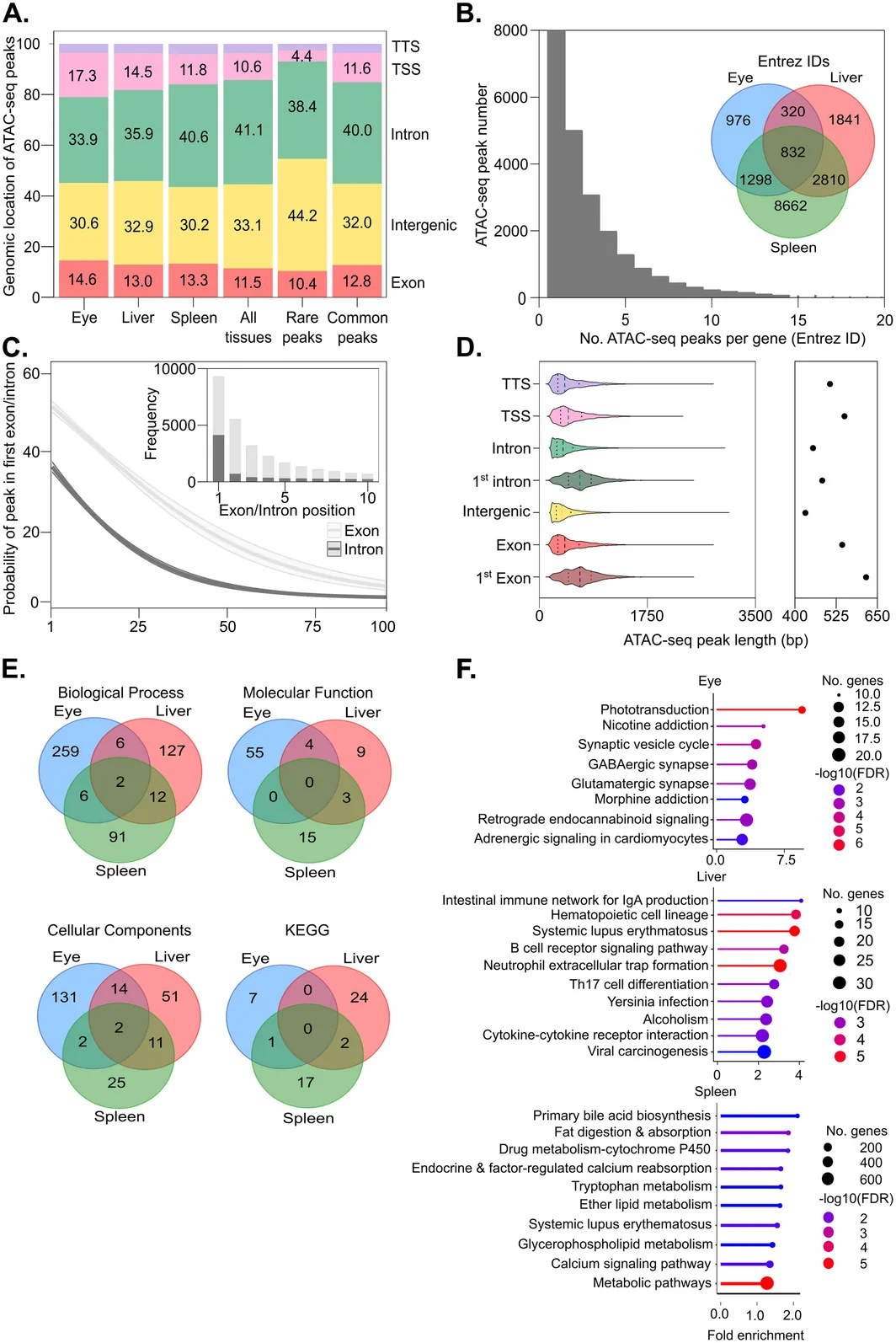
Functional annotation of ATAC‐seq peaks across tissues. (A) Relative proportion of annotated ATAC‐seq peaks across different tissues and peak categories. (B) Distribution of ATAC‐seq peaks assigned to *P. fluviatilis* genes (Entrez ID). Inset: Venn diagram showing shared and unique genes with assigned peaks among eye, liver and spleen. (C) Binomial mixed‐effect model of ATAC‐seq intron and exon data. The *y*‐axis shows the probability that a peak occurs in the first exon or intron; the total number of exons/introns per gene is displayed on the *x*‐axis. Inset: Density of ATAC‐seq peaks across relative exon/intron positions. (D) Left: ATAC‐seq peak lengths (bp) across annotation categories with quartile ranges (dotted lines) and median values (dashed lines). Right: Mean peak length (bp) across annotation categories. (E) Shared and unique GO terms between eye, liver and spleen samples across gene ontology categories. (F) Top 10 enriched KEGG pathways (FDR < 0.01) of genes associated with ATAC‐seq peaks observed in the eye, liver and spleen.

Across all tissues, ATAC‐seq peaks were predominantly located in intronic (31,421) and intergenic (25,270) regions, with fewer distributed in exonic, promoter‐TSS regions and TTS regions (Figure 2A). Rare peaks (occurring in 1–2 samples) showed a lower frequency at promoter–TSS regions (4.4%) compared to common peaks (11.6%). Tissue‐specific analysis revealed varying degrees of peak presence, with the spleen showing the highest number of accessible regions across all genomic features, followed by the liver, while eye tissue exhibited the fewest (Figure 2B inset). Most annotated genes were associated with a single (*n* = 8030) or fewer than five ATAC‐seq peaks (Figure 2B). Roughly 21% of the annotated genes (Entrez IDs) were shared among tissue types (Figure 2B inset).

Within genes, ATAC‐seq peaks showed preferential localization towards the beginning of genes, particularly within the first exon or first intron (Figure 2C). Because peaks were identified prior to annotation, feature length was not included as a covariate; this analysis evaluates the relative distribution of detected peaks among annotated gene features rather than the probability of peak detection as a function of feature size. The GLME showed that the probability of a peak being located in the first exon decreased with increasing total exon number (GLME: *β* = −0.0366, SE = 0.002, *z* = −15.80, *p* < 0.0001). A similar trend was observed for first introns, where the probability of a peak decreased with increasing intron count (GLME: *β* = −0.0546, SE = 0.002, *z* = −24.05, *p* < 0.0001). The model also revealed a sharp drop in probability after exon 1, while peaks in introns declined more gradually beyond intron 2 (Figure 2C inset). ATAC‐seq peak length distributions varied by annotation category; intronic (mean = 455 bp; SD = 245.7) and intergenic (mean = 432 bp; SD = 232.8) sites were the shortest categories, while exonic (mean = 543 bp; SD = 279.7) TSS (mean = 550 bp; SD = 288.9) and TTS (mean = 503 bp; SD = 280.0) peaks were slightly longer on average (Figure 2D).

Gene ontology and KEGG pathway enrichment analyses were performed to assess functional differences among tissues. In total, 198, 175 and 183 pathways were enriched (FDR < 0.01) in the eye, liver and spleen, respectively (Figure 2E; Table S3). However, overlap among tissues was minimal, with no KEGG pathways shared across all three tissues and at most two pathways overlapping between any pair of tissues (Figure 2F). This pattern indicates that most enriched pathways are tissue‐specific and likely reflect fundamental differences in regulatory programs driving tissue‐specific functions. In the eye, KEGG pathways were related to phototransduction and neurotransmission (e.g., glutamatergic and GABAergic synapses), as well as addiction‐related signalling, reflecting a high degree of neuronal specialization and synaptic activity (Figure 2F). In the liver, enriched KEGG pathways included immune‐related processes such as the intestinal immune network for IgA production and B cell receptor signalling, along with pathways associated with systemic diseases (e.g., systemic lupus erythematosus, viral carcinogenesis), highlighting the liver's immunological role and involvement in inflammation. In the spleen, enriched KEGG pathways included metabolic and signalling pathways, such as bile acid biosynthesis, tryptophan metabolism and calcium signalling, reflecting not only its role in immune regulation but also metabolism (Figure 2F). These findings may also point to regulatory tissue crosstalk, consistent with the emerging concept of the gut‐spleen‐liver axis, which has been recognized as an important regulator of host physiology, particularly in the context of immune interactions (Dey 2025).

### 3.2 Chromatin Accessibility Strongly Associates With Gene Expression

Across all three tissues, genes with ATAC‐seq peaks were significantly more likely to be expressed than genes lacking ATAC‐seq peaks (Fisher's exact test, adjusted *p* < 0.001; Table 2). In the spleen, genes with ATAC‐seq peaks were 10.28 times more likely to be expressed than genes without ATAC‐seq peaks (95% CI: 9.69–10.90). Similar effect sizes were observed in the liver (OR = 9.91, 95% CI: 9.36–10.50) and eye (OR = 9.20, 95% CI: 8.51–9.96). Consistent with this strong association, the majority of genes with ATAC‐seq peaks were expressed (81.6% in the spleen, 80.4% in the liver and 93.4% in eye). These results indicate a strong positive association between chromatin accessibility and transcriptional activity across the three tissues, consistent with the expected relationship between open chromatin and gene expression.

**TABLE 2:** Association between chromatin accessibility and gene expression across tissues.

Tissue	Expressed with ATAC‐peak	Expressed without ATAC‐peak	Not expressed, with ATAC‐peak	Not expressed, without ATAC‐peak	Odds ratio	95% CI	*p*	Bonferroni‐adjusted *p*
Spleen	13,756	2929	3104	6794	10.28	9.69–10.90	< 1E‐04	**< 1E‐04**
Liver	11,376	3644	2770	8793	9.91	9.36–10.50	< 1E‐04	**< 1E‐04**
Eye	11,701	8488	833	5561	9.20	8.51–9.96	< 1E‐04	**< 1E‐04**

*Note:* Counts of expressed and non‐expressed genes with and without overlapping ATAC‐seq peaks are shown for each tissue. Significant associations based on Fisher's exact test (Bonferroni corrected *p* < 0.05) are marked bold.

### 3.3 Variable Chromatin Accessibility and Differential Gene Expression

We then tested whether DEGs were more likely to be associated with variable chromatin accessibility compared to non‐DEGs (i.e., genes that are expressed, though not differentially). DEGs were enriched with variable chromatin regions (54.9%) compared to non‐DEGs (52.0%) in the spleen (Bonferroni‐adjusted *p*‐value = 0.015), but not in the eye (DEGs: 81.7%; non‐DEGs: 81.1%) or liver (DEGs: 61.4%; non‐DEGs: 60.0%) (Figure S2; Table S4). These results indicate that variation in open chromatin accessibility may play a role in driving the observed gene expression differences between humic‐ and clear‐water environments, although the effect was only detected in the specific tissue that exhibited the highest number of differentially expressed genes.

### 3.4 Enrichment of Transcription Factor Binding Sites in Open Chromatin

**FIGURE 3:**
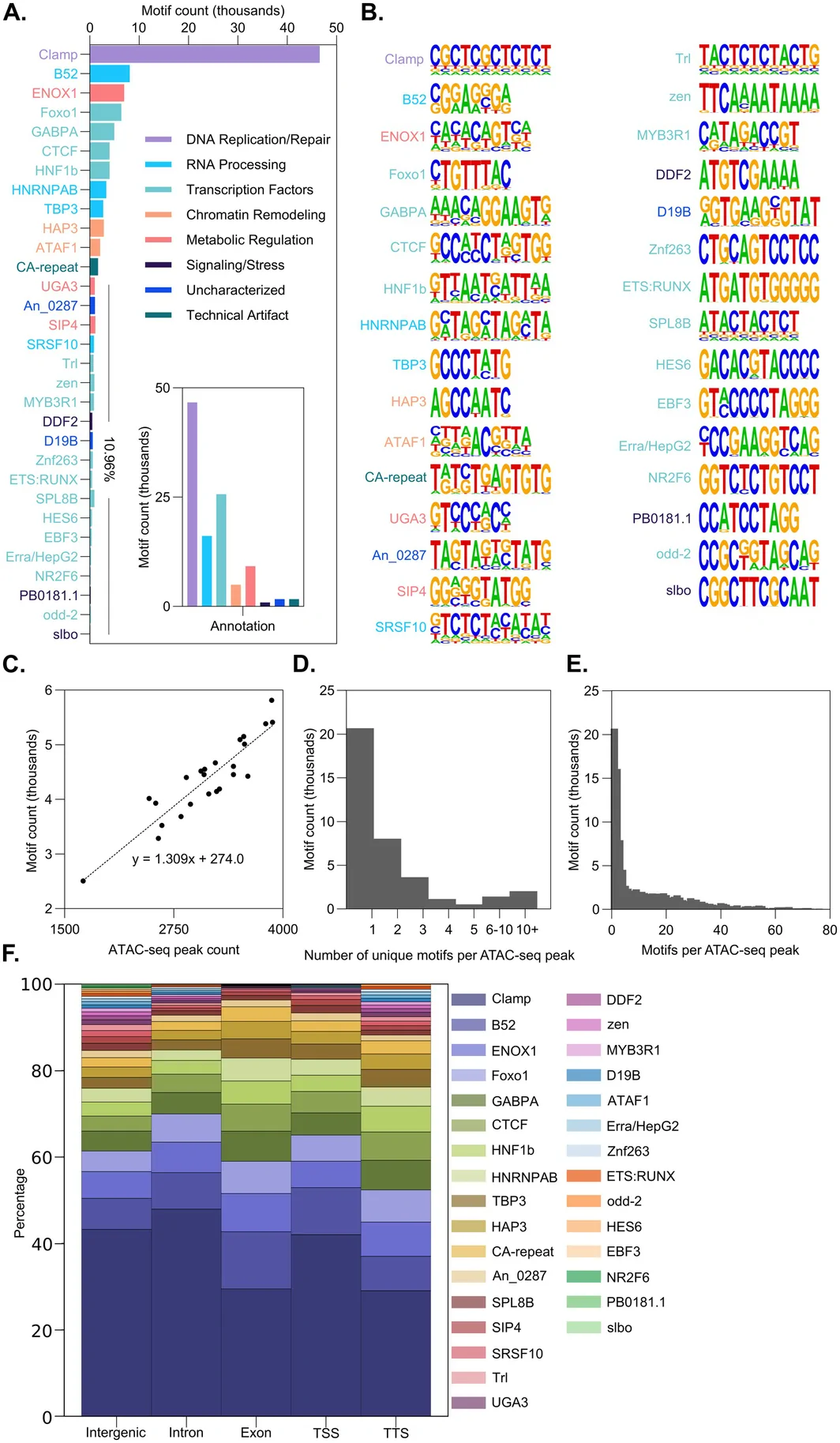
Enriched motifs within ATAC‐seq regions. (A) Frequency counts of 31 enriched motifs identified across all tissues. (B) Motif sequence logos representing the position weight matrices (PWMs) for the most frequent and high‐affinity transcription factors. (C) Correlation between motif count and ATAC‐seq peak count per chromosome. (D) Distribution of the number of unique motifs per ATAC‐seq peak. (E) Distribution of total motif occurrences per ATAC‐seq peak. (F) Relative frequency (%) of representative motifs across genomic annotation categories.

Exploration of motif occurrence revealed 31 highly enriched motifs across the three tissues (Figure 3A,B; Table S5). The most frequently occurring motifs included general regulatory factors such as Clamp (46,687 occurrences, 43.5%; mean score = 8.50), B52 (9170 occurrences, 8.54%; mean score = 7.64) and ENOX1 (7010 occurrences, 6.52%; mean score = 7.07). Notably, the highest affinity motifs were Znf263 (mean score = 16.03), EBF3 (mean score = 15.82) and HES6 (mean score = 15.82), though these exhibited lower occurrence frequencies (0.55%, 0.38% and 0.39%, respectively). We also identified several additional TF binding sites, including CTCF (4060 occurrences, 3.78%; mean score = 11.16, 5.10%), Foxo1 (6411 occurrences, 5.97%; mean score = 8.81), GABPA (5427 occurrences, 5.05%; mean score = 9.87) and HNF1b (4125 occurrences, 3.84%; mean score = 9.03).

These transcription factors are known to be involved in key processes such as chromatin organization (CTCF), stress response and metabolism (Foxo1), mitochondrial biogenesis and transcriptional activation (GABPA) and developmental regulation (HNF1b). Transcription factors such as FOXA1 act as ‘pioneer factors’, able to bind nucleosomal DNA and initiate chromatin remodelling; this enables binding by high‐affinity transcription factors and is crucial for cell‐type‐specific gene activation (Zaret and Carroll 2011). The analysis also revealed composite motifs—defined as two or more individual motifs that occur in close proximity—such as ETS:RUNX (475 occurrences, 0.44%; mean score = 15.72).

Functional annotation of enriched motifs revealed diverse roles, such as those linked to replication and repair (Clamp), while others were associated with RNA processing (e.g., B52, GABPA) and transcriptional regulation (e.g., Foxo1, ENOX1) (Figure 3A,B). Similar to ATAC‐seq peaks, the number of identified motifs scaled linearly with chromosome size (Figure 3C). Most peak regions contained a unique motif, with only a small minority containing five or more motifs (Figure 3D). The overall motif density per ATAC‐seq peak was relatively low, with one to four motifs per peak being the most common feature (Figure 3E). Motif distribution across genomic annotation categories (Figure 3F) revealed that B52, ENOX1, Foxo1 and GABPA were more frequent in exonic and intronic regions, while Clamp was consistently frequent across all annotation categories.

### 3.5 Tissue‐ and Habitat‐Associated Chromatin Accessibility Patterns

**FIGURE 4:**
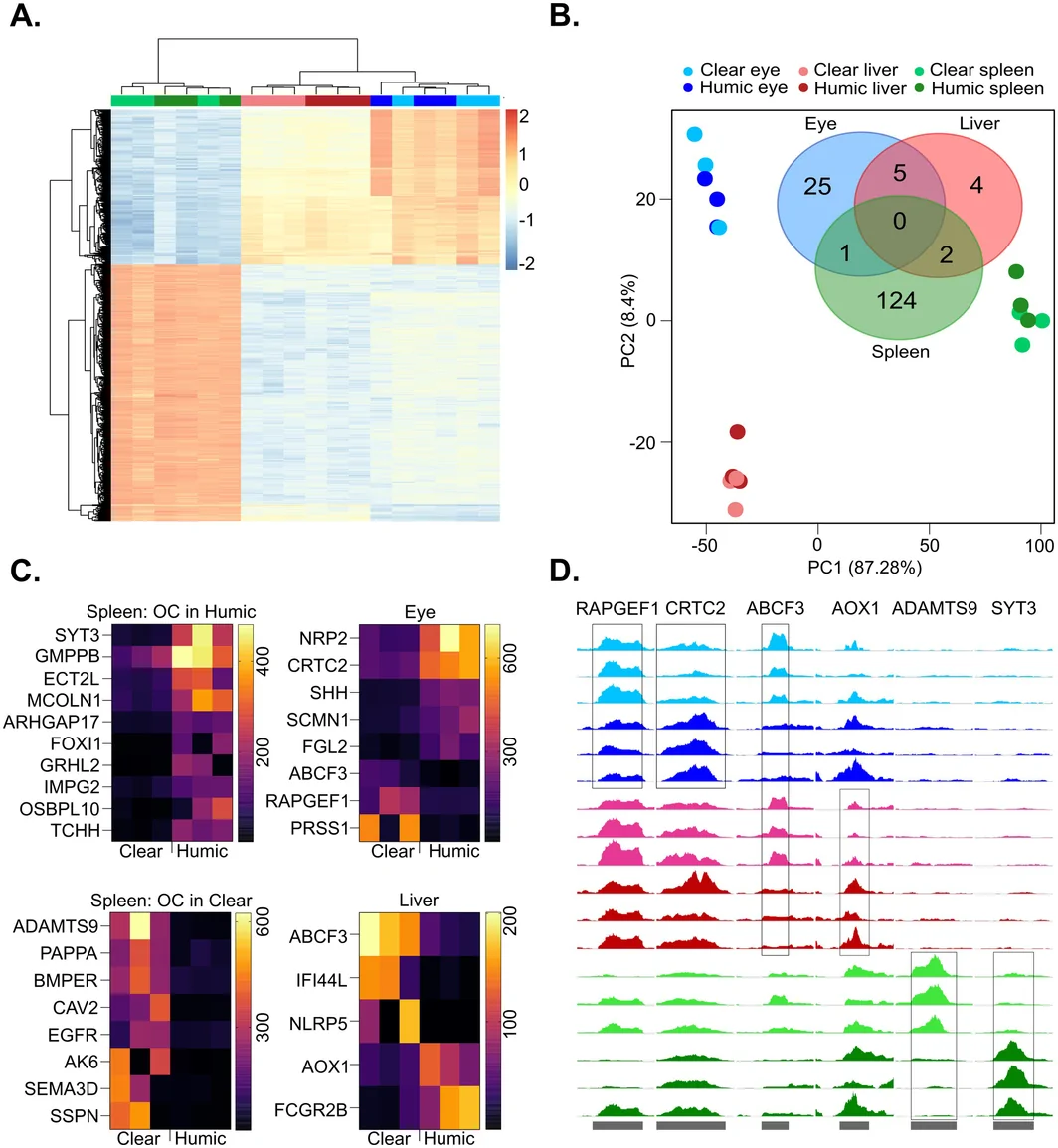
Differential chromatin accessibility across tissue types and environments. (A) Heatmap of the 76,850 ATAC‐seq peaks across all samples. Columns represent tissue samples (green: spleen; red: liver; blue: eye) and habitat condition (light colour: clear‐water; dark colour: humic‐water). (B) Multidimensional scaling (MDS) plot showing separation of samples by tissue type. Inset: Venn diagram of differentially accessible ATAC‐seq peaks between humic and clear‐water environments across tissues. (C) Heatmaps of representative differentially accessible ATAC‐seq peaks associated with humic‐ and clear‐water environmental differences (OC: Open Chromatin). (D) A subset of differentially accessible chromatin regions and genes associated with humic and clear‐water habitats in the three tissues. Boxes highlight regions of differential accessibility, and grey bars (bottom) correspond to the identified ATAC‐seq peaks.

We found strong tissue specificity, with samples clustering primarily by tissue type (spleen, liver and eye) rather than habitat type (Figure 4A). Multidimensional scaling (MDS) also supported distinct chromatin accessibility profiles across tissues (Figure 4B). Only 0.32% (*n* = 161) of ATAC‐seq peaks exhibited differential accessibility between humic‐ and clear‐water environments, with 124, 25 and four differentially accessible peaks detected in the spleen, eye and liver, respectively. Eight ATAC‐seq peaks showed environment‐dependent accessibility in at least two tissues (Figure 4B inset).

Differentially accessible peaks between humic‐ and clear‐water environments were located near genes with diverse biological roles. For example, the TCHH gene (PFLUV_G00086360), a gene which is associated with human hair and skin development (Medland et al. 2009; Wu et al. 2016), displayed markedly reduced chromatin accessibility in the spleen of clear‐water individuals compared to humic‐water individuals (Figure 4C). Environment‐dependent patterns were also found near vision‐related genes in the eye (Figure 4C,D), with particularly strong differences at the NRP2 (PFLUV_G00142740) and CRTC2 (PFLUV_G00090970) genes (Figure 4C), which are associated with axon guidance and energy metabolism, respectively (Schwarz et al. 2008; Hill et al. 2016). In addition, several ATAC‐seq peaks showed consistent accessibility changes across tissues, such as the ATP‐binding cassette gene ABCF3 (PFLUV_G00015820) and the cytosolic enzyme gene AOX1 (PFLUV_G00277450) in liver and eye tissue (Figure 4D).

### 3.6 Enrichment of Signatures of Selection in Open Chromatin Regions

In total, we found 186 outlier SNPs overlapping with ATAC‐seq peaks (Table 3). After Bonferroni correction for 19 independent tests, we did not observe significant enrichment of outliers within the global ATAC‐seq dataset. However, significant enrichment was observed within specific categories of open chromatin: ATAC‐seq peaks shared among 10–18 samples (defined as common peaks; adjusted *p* = 0.019) and peaks located at transcription start sites (TSSs; adjusted *p* = 0.019). These results suggest that certain regulatory regions, particularly TSSs, are more frequently targeted by natural selection than others such as intergenic or rare peaks. Tissue‐specific patterns also emerged, with the strongest enrichment of outliers observed in TSS peaks within individual tissues, reflecting their putative adaptive regulatory roles (Table 3).

**TABLE 3:** Enrichment of outlier SNPs in open chromatin regions compared to the genome‐wide data (López et al. 2025).

Category	No. of ATAC‐seq peaks	No. of ATAC‐seq peaks with SNPs	No. of SNPs within ATAC‐seq peaks	No. of outlier SNPs within ATAC‐seq peaks	No. of ATAC‐seq peaks with outlier SNPs	Frequency of outlier SNPs	Outlier enrichment test (unadjusted *p*)	Outlier enrichment test (Bonferroni adjusted *p*)	Outlier enrichment (fold change)
All samples	76,850	22,654	39,606	186	138	0.005	0.050	0.950	1.220
Rare peaks (1–2 samples)	24,814	4742	6958	21	19	0.003	0.175	1.000	0.732
Common peaks (10–18 samples)	16,128	6636	13,350	78	50	0.006	0.001	**0.019**	1.463
Not rare peaks (3–18 samples)	52,036	17,912	32,648	165	119	0.005	0.006	0.114	1.220
All intergenic	25,279	5593	10,036	29	18	0.003	0.065	1.000	0.732
All introns	31,420	9158	14,838	69	47	0.005	0.274	1.000	1.220
All exons	8767	3544	6334	38	31	0.006	0.017	0.323	1.463
All TSSs	8073	3282	6318	43	28	0.007	0.001	**0.019**	1.707
All TTSs	2831	1057	1854	7	6	0.004	0.850	1.000	0.980
Intron 1	9296	2021	5814	30	14	0.005	0.085	1.000	1.220
Other introns	22,124	4159	8987	32	23	0.004	0.475	1.000	0.976
Exon 1	4114	3761	1972	22	19	0.011	0.089	1.000	2.682
Other exons	4653	2573	1572	16	13	0.010	0.089	1.000	2.439
Present in eye	27,097	8789	16,563	100	70	0.006	1E‐04	**0.002**	1.463
Present in liver	37,669	11,201	21,174	105	75	0.005	0.041	0.779	1.220
Present in spleen	59,202	19,716	35,231	170	122	0.005	0.026	0.494	1.220
TSS eye	4635	2069	4225	34	20	0.008	5E‐05	**0.001**	1.951
TSS liver	5422	2409	4875	41	26	0.008	2E‐06	**3.8E‐05**	1.951
TSS spleen	6969	3021	5972	41	26	0.007	0.001	**0.019**	1.707
WGS Genome scan López et al. (2025)	—	—	—	—	—	0.0041	—		

*Note:* Significant enrichments (Bonferroni corrected *p* < 0.05) are marked bold. Bonferroni‐adjusted *p*‐values greater than 1 were set to 1.

### 3.7 Functional Annotation of Targets of Selection With Multi‐Omics

**FIGURE 5:**
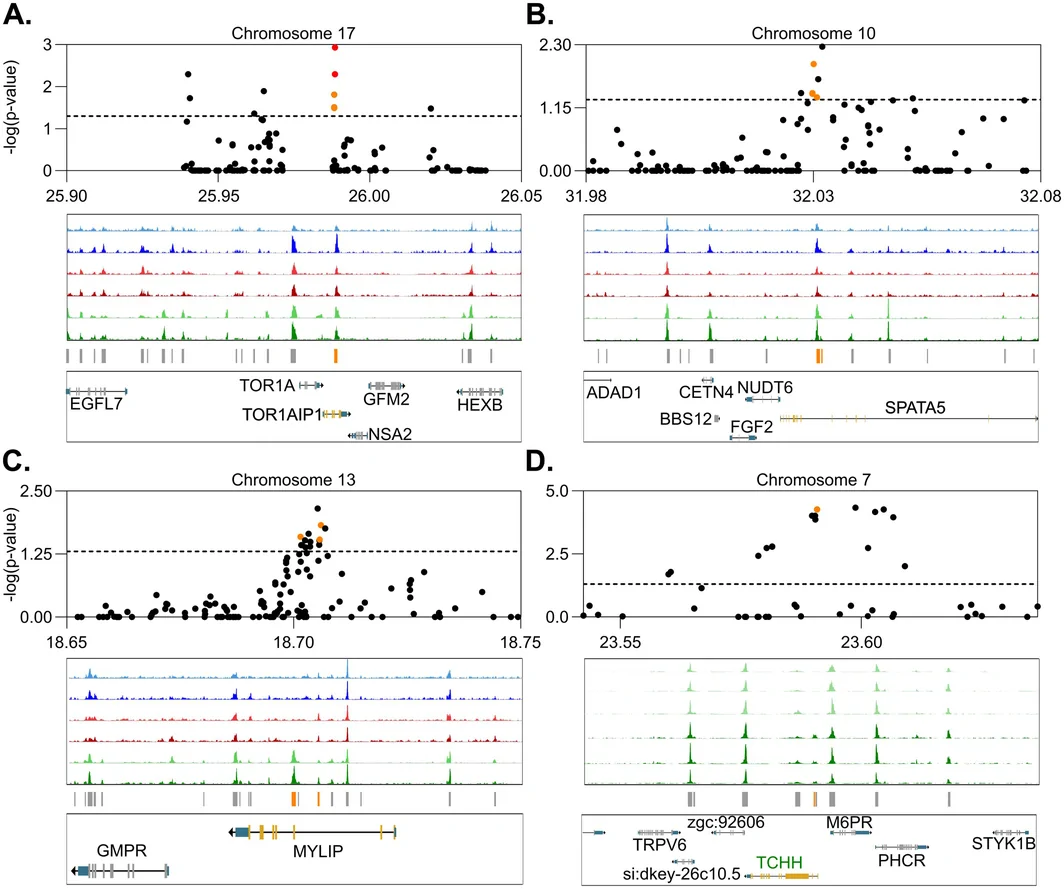
Overlap between outlier SNPs (López et al. 2025), open chromatin regions (ATAC‐seq), eQTLs and differentially expressed (DE) genes between humic and clear‐water environments (Ozerov et al. 2025). Genomic coordinates are shown in Mbp. (A–D) Four representative chromosomal regions containing candidate genes: (A) TOR1AIP1, (B) SPATA5, (C) MYLIP and (D) the TCHH gene. Top panels show −log_10_(*p*) values for individual SNPs; red indicates identified eQTL SNPs and orange points indicate outlier SNPs located within an ATAC‐seq peak. Bottom panels show ATAC‐seq signal tracks for a subset of samples (green: spleen; red: liver; blue: eye; light colour = clear‐water; dark colour = humic‐water), peaks are highlighted as grey bars and orange when harbouring outlier SNPs. The differentially expressed (DE) gene TCHH is highlighted in green to mark habitat‐specific expression divergence. Arrows in gene models indicate transcription orientation.

We evaluated four representative selective sweep genomic regions (Figure 5A–D), highlighting the potential regulatory relevance of open chromatin regions. These representative regions were selected based on their prior identification by Ozerov et al. (2025) as candidate genes under selection in either humic‐ or clear‐water perch and the notable presence of eQTLs. In the case of TOR1AIP1 (PFLUV_G00204080), we observed four tightly clustered outlier SNPs located within a single intronic ATAC‐seq peak (intron 1), two of which correspond to an identified eQTL for the olfactory rosette of *P. fluviatilis* (coloured in red). This supports the presence of a potential *cis*‐regulatory element within this intronic region that modulates expression variation (Figure 5A). In mice, TOR1AIP1 and its closely associated TOR1A gene have been linked to nuclear envelope integrity in neurons and synaptic transmission (Kim et al. 2010; Cossins et al. 2020). For SPATA5 (PFLUV_G00126700), three outlier SNPs were concentrated in a 500 bp stretch of open chromatin within a promoter‐TSS (Figure 5B), raising the possibility that these variants influence tissue‐specific promoter activity or disrupt transcription factor binding. MYLIP (PFLUV_G00156220) exhibited three outlier SNPs across two nearby accessible peaks (< 25 Kbp apart) (Figure 5C). Two of these SNPs mapped to an intergenic region upstream of the MYLIP gene, while the third fell within an intronic region (intron 2). Finally, for TCHH (PFLUV_G00086360), previously identified as a candidate gene under selection in humic‐water perch spleen (López et al. 2025; Ozerov et al. 2025), we found a clear ecological difference in chromatin accessibility: a differentially accessible first intronic peak, present in humic‐water populations but absent in clear‐water ones (Figure 5D, highlighted region). This pattern coincides with gene expression divergence in the spleen (Ozerov et al. 2025) and supports a putative role for chromatin regulation in local adaptation. Given the close proximity of some outlier SNPs within particular accessible regions (e.g., SPATA5, TOR1AIP1), these variants are likely to be linked and should be interpreted as candidate regulatory haplotypes rather than independent signals of selection. These four cases illustrate the added value of integrating multiple data types to identify candidate *cis*‐regulatory regions.

## 4 Discussion

Understanding the regulatory mechanisms underlying inter‐ and intra‐species diversity is central to evolutionary biology. Here, we used ATAC‐seq to characterize genome‐wide chromatin accessibility in the eye, liver and spleen tissues of the Eurasian perch, a common keystone freshwater fish. Our goal was to link regulatory variation to footprints of selection associated with contrasting humic‐ and clear‐water environments. We found that chromatin accessibility patterns were primarily tissue‐specific, localized to key genomic features such as promoters and were enriched for a diverse array of transcription factor binding motifs. Notably, open chromatin regions—specifically transcription start sites (TSSs)—were enriched for highly divergent SNPs associated with the humic–clear‐water contrast, supporting a role for regulatory elements in adaptive divergence. By first characterizing the regulatory landscape across tissues and then integrating these chromatin accessibility data with genomic regions shaped by divergent selection, eQTLs and differentially expressed genes, we identified several promising targets of selection. These findings highlight the power of multi‐omics approaches for dissecting the regulatory basis of local adaptation in natural populations.

We found that highly divergent outlier SNPs between humic‐ and clear‐water populations were not enriched across the full global set of ATAC‐seq peaks after Bonferroni correction. However, enrichment became apparent among open chromatin regions present in specific subsets of accessible regions, particularly in common ATAC‐seq peaks and regions overlapping with TSSs. TSSs are often associated with promoter activity and may represent hotspots of regulatory divergence (Tabach et al. 2007; Meers et al. 2018; Pettie et al. 2024). This aligns with growing evidence across taxa for the importance of regulatory evolution in adaptation (Wray 2007; Wittkopp and Kalay 2011; Signor and Nuzhdin 2018). For instance, in three‐spined stickleback ( *Gasterosteus aculeatus* ), ecotypic differences in chromatin accessibility and enhancers were observed between freshwater and marine populations (Verta and Jones 2019), suggesting that *cis*‐regulatory elements can act as precursors to adaptive evolution. Similarly, in *D. melanogaster* , population‐level genotypic variation has been shown to shape chromatin accessibility profiles, particularly in regions influencing gene expression (Huynh et al. 2023). In our study, despite pronounced tissue‐specific chromatin accessibility, divergent SNP enrichment was not consistently tissue‐specific. This suggests that selection acts primarily on shared accessible elements (e.g., TSSs) rather than on strictly tissue‐specific distal regulatory regions. Although regulatory regions emerged as candidate targets of selection in our study, different genomic features may contribute to adaptation through distinct mechanisms. For example, exonic variants can directly alter protein sequence and potentially affect protein function, whereas variants in promoters, TSS‐associated regions and other regulatory elements may influence gene expression without altering protein sequence (King and Wilson 1975; Wray 2007; Carroll 2008; Savisaar and Hurst 2018). In the context of humic‐ and clear‐water environments, regulatory variation may be particularly relevant because environmental differences may favour changes in gene regulation rather than alterations to protein‐coding sequences. Such regulatory changes could provide a mechanism for local adaptation by modulating physiological responses to contrasting environments. However, our results do not exclude contributions from protein‐coding variation, and adaptive divergence may involve both regulatory and coding changes.

Our ATAC‐seq data revealed a strong tissue‐specific regulatory architecture. Chromatin accessibility clustered primarily by tissue type rather than environment, consistent with the premise that regulatory landscapes are shaped predominantly by tissue identity. Similar results have been reported in mice and humans, where tissue type is a major determinant of the accessible chromatin landscape (Liu et al. 2019; Vierstra et al. 2020; The ENCODE Project Consortium et al. 2020). At the same time, evidence from other systems shows that environmental cues can modulate accessibility within specific tissues. For example, in the stickleback, single‐cell analyses of the pituitary demonstrated that chromatin accessibility is tightly coupled to gene expression under different photoperiods, highlighting environmentally responsive regulation in this key physiological tissue (Liu et al. 2025). Consistent with the regulatory role of open chromatin, genes overlapping ATAC‐seq peaks were substantially more likely to be transcriptionally active across all tissues, with odds ratios approaching tenfold enrichment. This strong association confirms that accessible chromatin regions correspond to functional regulatory elements influencing gene expression in *P. fluviatilis* . In contrast, few peaks (*n* = 161, 0.32%) exhibited differential accessibility between humic‐ and clear‐water environments in perch. These environmentally associated peaks could reflect either plastic responses to habitat conditions or genetic differences between populations, although our current design does not allow us to disentangle these effects. Such ambiguity has been observed in other studies, where both genotype and environment influence chromatin accessibility (Meuleman et al. 2020; Huynh et al. 2023).

Despite environment‐associated peaks being infrequent, several were located near genes with plausible functional relevance. For instance, in the eye, we observed increased accessibility in humic‐water individuals at peaks near NRP2 and CRTC2 genes, which are associated with axon guidance and metabolic stress response, respectively (Islam et al. 2022; Han et al. 2018). In the liver, differences in accessibility near ABCF3 and AOX1 genes, associated with immune defence and energy metabolism, may reflect habitat‐specific adaptation. ABCF3‐associated peaks were more accessible in clear‐water perch. This gene has been shown to interact with OAS1B and is required for resistance to flavivirus infection in mice, indicating a function in antiviral immunity (Courtney et al. 2012; Peterson et al. 2019). In contrast, AOX1 peaks were more accessible in humic‐water perch, and AOX1 (aldehyde oxidase 1) is linked to mitochondrial function and broader energy metabolism, and changes in its regulation could influence metabolic adaptation (Garattini et al. 2009). While these accessibility changes do not necessarily guarantee downstream transcriptional effects, their proximity to genes with environmentally relevant functions is suggestive of a potential regulatory role in adaptation or acclimatization.

Previous studies have shown that accessible chromatin regions can regulate gene expression in a context‐dependent manner, requiring specific transcription factor binding or chromatin remodelling for functional impact (Heinz et al. 2015; Nair et al. 2019; Nair et al. 2024). Our findings show that only 0.32% of the peaks differed between humic‐ and clear‐water environments, which may suggest that environmentally responsive regulation occurs through subtle, region‐specific changes rather than widespread chromatin shifts. Importantly, these subtle differences are enriched in genomic regions previously identified by López et al. (2025) as being highly divergent between humic‐ and clear‐water environments, providing evidence for an adaptive basis. However, this contrasts with results in dairy calves experimentally infected with bovine respiratory syncytial virus, where ATAC‐seq analysis identified roughly between 13%–17% of peaks as differentially accessible in bronchial lymph nodes, suggesting a strong chromatin remodelling response to severe infection (Johnston et al. 2021). More extreme patterns have also been observed in other taxa. For instance, in the silverleaf whitefly ( *Bemisia tabaci* ), temperature stress induced reproducible changes in chromatin accessibility, particularly near genes related to the stress response and metabolism (Shen et al. 2023). In the sea anemone ( *Nematostella vectensis* ), heat exposure elicited differential accessibility that depended on environmental conditioning history, underscoring the interplay between exposure history and chromatin state (Weizman et al. 2021). These examples highlight that while environment‐induced chromatin changes can be dramatic under acute or extreme conditions, the localized differences we observe in perch may reflect fine‐scale regulatory adaptation in natural populations rather than broad stress responses.

Motif analysis provided further insight into the regulatory landscape. We identified 31 enriched motifs, including broadly acting regulatory factors such as Clamp, B52 and ENOX1, as well as high‐affinity motifs like GABPA and CTCF, known for their roles in metabolic regulation and chromatin architecture, respectively (Holwerda and de Laat 2013; Yang et al. 2014; Kadota et al. 2017; Gu et al. 2018; Kentepozidou et al. 2020; Wilson et al. 2020). Many of the enriched motifs correspond to transcription factors implicated in the stress response, immune regulation and metabolic adaptation, biological processes previously identified as targets of selection in the humic adaptation of *P. fluviatilis* (Ozerov et al. 2022, 2025). These motifs were non‐randomly distributed across annotation categories, with peaks enriched for both high‐frequency motifs like Clamp and B52 and high‐affinity motifs like GABPA and CTCF, consistent with previous findings in both the zebrafish and stickleback (Voss et al. 2011; Verta and Jones 2019). Of these, GABPA plays a central role in regulating mitochondrial biogenesis and oxidative metabolism, processes that may be essential for tolerating humic‐associated stressors such as low oxygen and low pH (Yang et al. 2014; Wilson et al. 2020). The recurrent enrichment of CTCF, a master regulator of chromatin structuring and transcription insulation (i.e., blocking inappropriate interactions between enhancers and promoters), has been implicated in enabling environmentally responsive gene expression programs in both mammals and fish (Holwerda and de Laat 2013; Kadota et al. 2017; Gu et al. 2018; Kentepozidou et al. 2020). Its presence across tissues and annotation categories suggests a conserved role in anchoring regulatory regions, which modulate chromatin structure and transcription under varying environmental conditions. The Clamp motif, an element that allows sliding clamps to attach to DNA for the purpose of replication and repair (Simonsen et al. 2024), is also widely conserved across species and has been shown to modulate chromatin accessibility and transcription in promoter regions, as studied in *Drosophila* (Kuzu et al. 2016); it may play a similar role within the regulatory landscape of *P. fluvatilis* across tissues. Motifs like B52 are involved in alternative splicing regulation and have been shown to modulate splicing patterns under stress in *Drosophila* as well (Shi et al. 1997; Juge et al. 2010). While transcription factors like Clamp provide access to transcriptional machinery, rarer high‐affinity motifs are likely to mediate specific regulatory interactions. Although suggestive, the co‐occurrence of high‐frequency motifs (e.g., Clamp, B52) with rarer high‐affinity motifs (e.g., Znf263, EBF3) may reflect a two‐tiered regulatory mechanism (Shahein et al. 2022). Together, these patterns suggest that a core set of regulatory elements, conserved across tissues, interacts with the chromatin landscape to enable flexible gene regulation.

GO enrichment of accessible chromatin regions highlighted strong tissue‐specific regulatory landscapes, consistent with the distinct physiological roles of the eye, liver and spleen. In the eye, enrichment for phototransduction and neurotransmission pathways aligns with known sensory specialization in fish visual systems (Lu et al. 2024). Similarly, in the liver, accessibility at immune‐ and inflammation‐related regions supports its well‐established role in immune–metabolic functions, particularly in response to stressors (Iwama et al. 2006; Liu et al. 2014; Birnie‐Gauvin et al. 2017; Kubes and Jenne 2018; Zhou et al. 2022). In the spleen, enrichment for metabolic functions further emphasizes its dual role in immune surveillance and metabolic regulation (Wan et al. 2024). The minimal overlap of enriched pathways across tissues underscores the value of multi‐tissue approaches, which reflect the breadth of regulatory functions that may be overlooked in single‐tissue studies.

By integrating ATAC‐seq with transcriptomic (RNA‐seq, eQTL; Ozerov et al. 2025) and population genomic (Pool‐seq; López et al. 2025) datasets, we identified regulatory regions and variants with potential adaptive significance. We focused on four representative genomic regions, which were selected based on prior research identifying these genes as divergent between environments (Ozerov et al. 2025; López et al. 2025) and/or based on the overlap of multiple data layers associated with divergence between humic‐ and clear‐water environments. In several regions, selection signals spanned multiple genes. At the TOR1AIP1 locus (chromosome 17), an intronic ATAC‐seq peak co‐localized with both an eQTL and multiple outlier SNPs, strengthening the case that this gene, rather than neighbouring ones, is a functional target of selection. Additionally, TOR1AIP1 showed environment‐associated differences in chromatin accessibility specifically in the eye tissue. This gene plays a role in nuclear envelope stability and neuronal signalling (Kim et al. 2010; Cossins et al. 2020; Mackels et al. 2023), suggesting it may be involved in sensory or stress‐related regulatory adaptation. A similar pattern emerged at the SPATA5 locus (chromosome 5), where outlier SNPs overlapped with an ATAC‐seq peak located in the promoter and transcription start site (promoter–TSS) region. Additional peaks were also observed in intronic regions, indicating potential regulatory activity across multiple gene regions. SPATA5 is involved in mitochondrial function and has been linked to neurodevelopmental and visual disorders (Tanaka et al. 2015; Buchert et al. 2016), raising the possibility that environment‐driven changes in accessibility could impact energy metabolism or sensory traits. MYLIP (chromosome 13), a gene involved in hypoxia and immune regulation (Li et al. 2025), displayed ATAC‐seq peaks in both intergenic and intronic regions, with tissue‐specific accessibility most pronounced in the spleen, regardless of environment. Notably, MYLIP was one of the top genome‐wide selection candidate genes identified by Ozerov et al. (2022) between clear‐ and humic‐water Eurasian perch, with pronounced allele frequency differences between these populations. This gene was further identified as a candidate of selection by López et al. (2025). MYLIP has been implicated in multicellular development, lipid metabolism, plasma membrane‐bound cell projection organization, and nervous system regulation (Olsson et al. 1999; Bornhauser et al. 2003; Lindholm et al. 2009; Calkin et al. 2011; van Loon et al. 2019), indicating that selection at this locus may reflect broader physiological adaptation to humic lake environments. Finally, at the TCHH locus (chromosome 7), an intronic (intron 2) ATAC‐seq peak uniquely accessible in humic environments contained outlier SNPs. This gene, known for its role in epithelial structural integrity, was previously found to be upregulated in humic perch (Ozerov et al. 2025), suggesting that chromatin‐mediated regulation at this locus may enhance epidermal resilience under acidic environmental conditions. Together, these case studies demonstrate how multi‐omic integration, linking chromatin accessibility with expression data and population genetic signals, can help prioritize candidate regulatory elements for functional follow‐up in natural populations (Mack et al. 2018; Artemov et al. 2017; Li et al. 2023; Yuan et al. 2023; Aramburu et al. 2025; Wenz et al. 2025; Wong and Tremethick 2024).

Our study also has some important limitations. The modest sample size (18 samples, all females) and the use of a single time point restricts our ability to capture fine‐scale variation or sex‐ and season‐specific regulatory dynamics. This limited ATAC‐seq sample size also reduces statistical power to detect subtle differences in chromatin accessibility; consequently, some environmentally associated regulatory changes may have remained undetected. Therefore, our analyses should be interpreted as a set of robust candidate accessibility changes rather than an exhaustive assessment of all regulatory differences between humic‐ and clear‐water populations. Moreover, while we detect environmentally associated differences in accessibility, the present design cannot fully separate environmentally induced plasticity from underlying genetic divergence (e.g., Davidson and Moczek 2024; Nobrega et al. 2024). ATAC‐seq identifies putative regulatory elements but does not directly establish causal function. Future work expanding sampling across sexes, seasons, sample size and additional tissues (e.g., gill, brain), coupled with allele‐specific accessibility analyses and functional assays, will be crucial to validate the regulatory activity of candidate loci and to empower more robust genome‐wide analyses of accessibility–expression relationships. Larger studies, as demonstrated in humans, have revealed widespread genetic regulatory mechanisms through chromatin availability (Wenz et al. 2025), underscoring the importance of increasing sample size and diversity in future ecological genomics studies. Such approaches will help bridge the gap between association and mechanism, providing deeper insight into the role of chromatin regulation in local adaptation. Nevertheless, by combining ATAC‐seq with transcriptomic and population genomic data in a wild vertebrate, our study already provides a strong foundation for linking chromatin accessibility to adaptive divergence in natural populations.

This study is among the first to apply ATAC‐seq to a wild vertebrate species, providing a comprehensive view of the regulatory landscape in a keystone freshwater fish. Our results demonstrate that open chromatin regions, especially those near TSSs, are enriched for divergent SNPs, suggesting that regulatory regions represent frequent targets of selection. While environmental differences had a modest effect on chromatin accessibility, tissue‐specific patterns revealed alignment with known physiological functions, supporting the role of regulatory architecture in shaping organ‐specific expression. Furthermore, overlapping ATAC‐seq data with differential gene expression and eQTL data highlighted promising candidate genes and putative causal variants for adaptive regulatory divergence. Importantly, this study's multi‐layered integration of chromatin accessibility, population genomic signals and transcriptomic data allows us to identify regulatory mechanisms with putative adaptive significance. Such integrative analyses remain rare in wild non‐model populations and provide a basis for prioritizing candidate loci for future functional analyses. Together, these findings establish a functional framework for linking population genomic signals to regulatory mechanisms and lay the groundwork for the experimental validation of adaptive variants in natural populations.

## Author Contributions

Anti Vasemägi and Kristina Noreikiene devised and planned the study. Anti Vasemägi and Kristina Noreikiene conducted field sampling. Konrad Taube conducted bioinformatics analyses. María‐Eugenia López assisted with bioinformatic analyses. Konrad Taube, Anti Vasemägi, and Kristina Noreikiene collaborated in writing the initial draft of the manuscript. Riho Gross and María‐Eugenia López contributed to manuscript editing. All authors participated in revising and refining the manuscript.

## Funding

This study was supported by the Swedish Research Council (grant 2020‐03916 to Anti Vasemägi) and the Estonian Research Council (PRG852 to Riho Gross).

## Ethics Statement

A total of six female *P. fluviatilis* individuals were collected from three clear‐ and three humic‐water lakes in Estonia in August 2021 using gillnets and rods. Samples were collected in accordance with national legislation based on permit issued by the Estonian Ministry of Environment (no. 10‐1/21/18). Fish were euthanized with an overdose of benzocaine and measured for wet weight and total length. Ethical aspects of sampling were conducted according to the requirements of Annex IV (Methods of killing animals) Section B point 11 of the Directive 2010/63/EU of the European Parliament and of the Council of 22 September 2010 on the protection of animals used for scientific purposes. Specific ethical permit for sampling of fish is not required in Estonia.

## Conflicts of Interest

The authors declare no conflicts of interest.

## Supporting information


**Figure S1:** Sampling map of *P. fluviatilis* in Estonia. Clear‐ and humic‐water lakes are displayed with unfilled and filled circles, respectively.
**Figure S2:** Venn diagrams illustrating the overlap between genes with peaks and genes classified as expressed (from Ozerov et al. 2025) in eye, liver and spleen. Numbers indicate the counts of genes unique to each category and shared between the expressed (left) and peak‐associated (right) gene sets.

**Table S1:** Metadata and sequencing statistics for ATAC‐seq samples, including the collection location (lake name, type and GPS coordinates), sample identifiers, raw sequencing metrics (total reads, paired‐end read counts and percentage of properly paired reads) and results from peak calling (number of identified ATAC‐seq peaks).
**Table S2:** Consensus ATAC‐seq peak data, including genomic coordinates, sub‐peak presence across samples, functional annotations and sample‐specific read counts (featureCounts). In G‐X, ✓ indicates sample presence in the peak, while X indicates sample absence.
**Table S3:** Summary of GO terms enriched for each tissue type (FDR < 0.01). Biological processes (BP), cellular components (CC), molecular function (MF) and Kyoto Encyclopedia of Genes and Genomes (KEGG) have been included.
**Table S4:** Association between variable chromatin accessibility and differential gene expression across tissues. Significant association based on Fisher's exact test (Bonferroni‐adjusted *p* < 0.05) is marked bold. Non‐significant associations are shown as ns.
**Table S5:** Annotated motif data for consensus ATAC‐seq peaks. Columns include peak ID, genomic coordinates, functional annotation, distance to TSS, CpG density, GC content and predicted transcription factor motifs with associated metadata (predicted transcription factor, database match, similarity score (in parenthesis), distance from peak center, sequence, strand and conservation).

## Data Availability

The ATAC‐seq data used in this study was submitted to the SRA (https://www.ncbi.nlm.nih.gov/sra) with BioProject accession PRJNA1336071. Genome browser files, and large Tables S1–S5 have been submitted to figshare with DOI: 10.6084/m9.figshare.30272389, and custom scripts have been submitted to figshare with DOI: 10.6084/m9.figshare.31970346.
