# Supplementary material for: Chromatin Accessibility Reveals Adaptive Regulatory Variation in a Keystone Freshwater Fish
Source: Mol Ecol. 2026 Sep 21;35(18):e70550. doi: 10.1111/mec.70550 (PMC13594500; doi:10.1111/mec.70550)
Supplement: Supplementary file 1 [file MEC-35-e70550-s001.docx]

*Supplementary figures for*:

**Chromatin accessibility reveals adaptive regulatory variation in a keystone freshwater fish**

Konrad Taube^1^, Kristina Noreikiene^2^, María-Eugenia López^3^, Riho Gross^1^, Anti Vasemägi^1,3^

^1^Estonian University of Life Sciences, ^2^Vilnius University, ^3^Swedish University of Agricultural Sciences

**Corresponding author**: Anti Vasemägi, Sötvattenslaboratoriet, Stångholmsvägen 2, 17893 Drottningholm, anti.vasemagi@slu.se

**Supplementary figures S1-S2**

**Fig. S1.** Sampling map of P. fluviatilis in Estonia. Clear- and humic-water lakes are displayed with unfilled and filled circles, respectively.

**Fig. S2.** Venn diagrams illustrating the overlap between genes with peaks and genes classified as expressed (from Ozerov et al., 2025) in eye, liver, and spleen. Numbers indicate the counts of genes unique to each category and shared between expressed (left) and peak-associated (right) gene sets.
